# Impact of the sequence of system-environment interactions on the functionality and efficiency of quantum thermal machines

**DOI:** 10.1038/s41598-025-95330-1

**Published:** 2025-04-01

**Authors:** Rui Huang, Zhong-Xiao Man, Lu Li, Yun-Jie Xia

**Affiliations:** School of Physics and Physical Engineering, Qufu Normal University, Qufu, 273165 China

**Keywords:** Quantum thermodynamics, Quantum thermal machine, Cascaded model, Collision model, Quantum information, Thermodynamics

## Abstract

In this work, we investigate effects of the sequence of system-environment interactions on the functionality and performance of quantum thermal machines (QTMs). The working substance of our setup consists of two subsystems, each independently coupled to its local thermal reservoir and further interconnected with a common reservoir in a cascaded manner. We demonstrate the impact of the sequential interactions between the subsystems and the common reservoir by exchanging the temperatures of the two local reservoirs. Our findings reveal that, when the two subsystems are in resonance, such an exchange alters the efficiency of QTMs without changing their functional types. Conversely, when the two subsystems are detuned, this exchange not only changes the efficiency but also the types of QTMs. Our results indicate that the manners of system-reservoir interactions offer significant potential for designing QTMs with tailored functionalities and enhanced performance.

## Introduction

With the progress in quantum information science and technology, it has become increasingly clear that quantum mechanics offers unique resources and effects that allow for the achievement of tasks that are unattainable through classical resources and traditional approaches^1^. Consequently, the extension of the benefits of quantum resources and effects into various traditional domains has become as a crucial and pertinent topic. In this regard, quantum thermodynamics, which integrates classical thermodynamics with quantum mechanics, has garnered significant attention^2–7^. One of its primary goals is to explore the application of quantum tools in the design of quantum thermal machines (QTMs)^8–10^, thereby demonstrating the quantum superiority within the realm of thermodynamics.

The operating prototype of the QTM was first conceptualized by Scovil and Schultz-Dubois^11^, paving the way for subsequent proposals of a multitude of QTM models. QTMs adopt quantum systems as their working material to accomplish thermodynamic tasks through interaction with thermal baths at different temperatures, occasionally with the assistance of external power sources. In general, QTMs can be classified into two categories: stroke-based and continuous ones. Stroke-based QTMs encompass several independent heat and power processes, such as traditional Otto and Carnot cycles^12–22^, as well as simplified versions that operate with just two strokes^23,24^. On the other hand, in continuous QTMs, the working material remains continually coupled with thermal baths to perform thermodynamic tasks^25–27^. One of the primary ways to utilize quantum resources and effects in actual QTMs is to incorporate them into the working substance^28–40^. Research has demonstrated that the quantum refrigerator comprising just three qubits can surpass its classical counterpart in terms of cooling efficiency and energy transfer, thanks to the entanglement of these qubits^28^. Furthermore, quantum engines employing two coupled qubits to execute a generalized Otto cycle exhibit a link between the work produced and the qubits’ correlations^29^. Experimental results also support the superiority of quantum resources in powering quantum engines, exemplified by the utilization of nitrogen vacancy centers in diamond^41^ and a spin-1/2 system with nuclear magnetic resonance techniques^32^.

In comparison to QTMs operating on a stroke-based mechanism, the continuous QTMs avoid the need for stroke transformations, resulting in a simpler design, which is also the focus of this work. A paradigmatic illustration of continuous QTM consists of coupled systems interacting at their boundaries with two reservoirs of different temperatures, which is also referred to as boundary-driven QTMs^42–47^. If the internal interaction of the system cannot satisfy energy conservation, the involvement of external work source is necessary, enabling it to function as an engine, refrigerator, or accelerator^42^. This boundary-driven two-reservoir model has been extended to the situation with three reservoirs, where an extra common reservoir is introduced for the whole system apart from the two local ones^48–51^. It turns out that due to the unique quantum effects brought about by the common reservoir, QTMs can achieve additional functionalities and experience performance enhancements. It shows that the machine can operate in otherwise forbidden regimes in the presence of a common non-equilibrium reservoir with coherence^50^. By considering a bipartite system coupled to both independent and common reservoirs, the effects of different types of system-reservoir interactions on work costs are unveiled and the operating regimes of QTMs are manifested^51^.

**Fig. 1:**
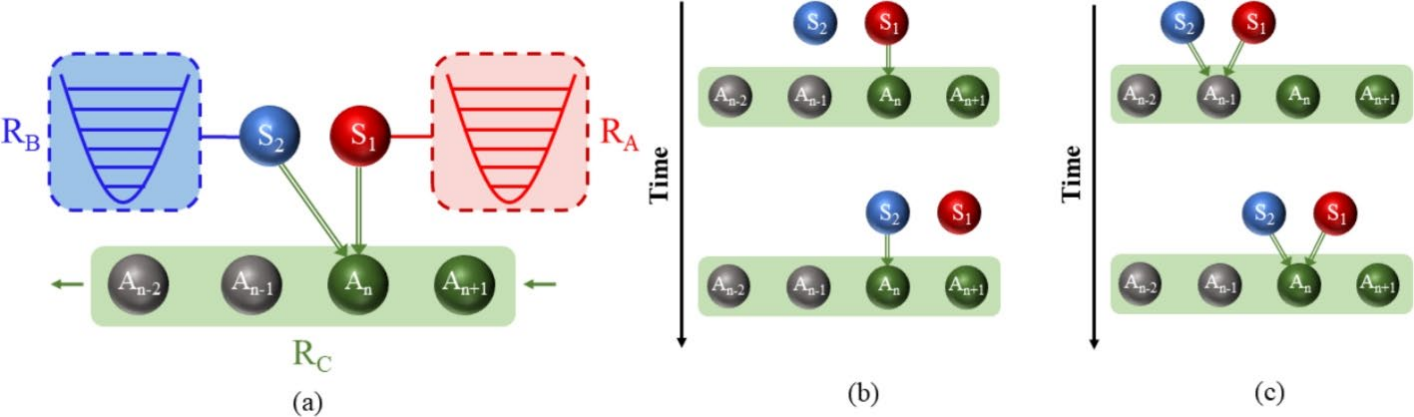
(Color online) (**a**) Schematic diagram of our setup. The working substances $S_{1}$ and $S_{2}$ are locally coupled to the thermal reservoirs $R_A$ and $R_B$, respectively, and meanwhile interact with the third reservoir $R_{C}$. Within the framework of collision model, the reservoir $R_{C}$ is simulated by a collection of identically prepared ancillae. (**b**) Schematic diagram of the cascade model, where the subsystem $S_1$ interacts with a generic ancilla in $R_{C}$ first, followed by the interaction of $S_2$ with the same ancilla. (**c**) Schematic diagram for the simultaneous interaction between $S_1$ and $S_2$ with a generic ancilla.

In addition to simultaneous interactions with a common reservoir, subsystems of a multipartite system can interact with the reservoir sequentially, a configuration known as the cascade model^52–55^. The impact of cascaded model on QTMs is an issue worthy of exploration. The cascaded model illustrates a scenario where subsystems, specifically $S_{1}$ and $S_{2}$, successively interact with a reservoir in such a way that $S_{1}$ interacts with it at first, followed by $S_{2}$, resulting in a unidirectional influence from $S_{1}$ to $S_{2}$. This model possesses an inherent temporal structure, enabling it to delineate the sequential interactions between subsystems and the reservoir. For example, in a linear arrangement of QED cavities, information (or energy) can be exchanged unidirectionally through the successive passage of injected atoms through each cavity. The question of how cascaded interactions impact the functionality and performance of QTMs, in contrast to simultaneous interactions, remains unresolved. In this study, we investigate a QTM composed of two subsystems, each coupled to its own local reservoir and, concurrently, interacting with a third reservoir in a cascaded fashion. For comparative analysis, we also explore the scenario where the two subsystems interact simultaneously with the third reservoir. Our primary focus is on the operational regimes attainable through these models, with an aim to identify configurations that could potentially enhance the performance of QTMs.

## The model and master equation

Our system $\mathcal {S}$ consists of two subsystems $S_{1}$ and $S_{2}$ being coupled locally to their own reservoirs $R_{A}$ and $R_{B}$, respectively. The two subsystems are bridged by the third reservoir $R_{C}$, with which they interact together (cf. Fig.1a). Within the framework of collision model^56–64^, the reservoir $R_{C}$ is simulated as a collection of identically prepared ancillae with the generic Hamiltonian $\hat{H}_{R_{C}}$. The system interacts/collides with the ancillae one by one, with each collision lasting a short duration $\tau$. After each collision, the ancilla is discarded, and the system collides with a new one in the next step of collision. The cascaded model (cf. Fig.1b) represents a unique type of collision model, wherein the subsystem $S_{1}$ collides firstly with an ancilla, followed by $S_{2}$ colliding with the same ancilla. Consequently, the dynamics of $S_{1}$ exert an influence on $S_{2}$, but the reverse does not hold true. For comparison, we also consider the simultaneous interactions between the system, $S_{1}$ and $S_{2}$, and the ancilla (cf. Fig.1c).

To be specific, we assume that both the subsystems $S_{1}$ and $S_{2}$ and generic ancilla of the reservoir $R_{C}$ are two-level systems with the Hamiltonians $\hat{H}_{S_{1}}=\omega _{S_{1}}\hat{\sigma }^{z}_{S_1}/2$, $\hat{H}_{S_{2}}=\omega _{S_{2}}\hat{\sigma }^{z}_{S_2}/2$ and $\hat{H}_{R_{C}}=\omega _{C}\hat{\sigma }^{z}_{R_{C}}/2$, respectively, in which $\omega _{S_{1}}$, $\omega _{S_{2}}$ and $\omega _{C}$ are the corresponding frequencies and $\{\hat{\sigma }^{x}_{\mathcal {O}},\hat{\sigma }^{y}_{\mathcal {O}},\hat{\sigma }^{z}_{\mathcal {O}}\}$ the usual Pauli operators acting on $\mathcal {O}$. The local reservoirs $R_{A}$ and $R_{B}$ are considered to be Bosonic ones governed by $\hat{H}_{R_{A}}=\sum _{k}\omega _{A,k}\hat{a}_{k}^{\dag }\hat{a}_{k}$ and $\hat{H}_{R_{B}}=\sum _{j}\omega _{B,j}\hat{b}_{j}^{\dag }\hat{b}_{j}$, respectively, with $\omega _{A,k}$ ($\omega _{B,j}$) the frequency of mode *k* (*j*) of reservoir $R_{A}$ ($R_{B}$), $\hat{a}^{\dag }_{k}$ ($\hat{b}^{\dag }_{j}$) and $\hat{a}_{k}$ ($\hat{b}_{j}$) are annihilation and generation operators of mode *k* (*j*). The local interactions of $S_{1}-R_{A}$ and $S_{2}-R_{B}$ are characterized by the Hamiltonians

1 $$\begin{aligned} \hat{H}_{S_{1}R_{A}}=\sum _{k}g_{A,k}\left( \hat{\sigma }^{+}_{1}\hat{a}_{k} +\hat{\sigma }_{1}^{-}\hat{a}^{\dag }_{k}\right) , \end{aligned}$$

and

2 $$\begin{aligned} \hat{H}_{S_{2}R_{B}}=\sum _{j}g_{B,j}\left( \hat{\sigma }^{+}_{2}\hat{b}_{j} +\hat{\sigma }_{2}^{-}\hat{b}^{\dag }_{j}\right) , \end{aligned}$$

with $g_{A,k}$ and $g_{B,j}$ the coupling strengths, and $\hat{\sigma }^{+}_{1}$ ($\hat{\sigma }^{+}_{2}$) and $\hat{\sigma }^{-}_{1}$ ($\hat{\sigma }^{-}_{2}$) the raising and lowering operators for the two-level subsystem $S_{1}$ ($S_{2}$), respectively.

The total Hamiltonian of the system plus reservoirs can be expressed as

3 $$\begin{aligned} \hat{H}_{tot}(t)=\hat{H}_{S}+\hat{H}_{R}+\hat{H}_{S_{1}R_{A}}+\hat{H}_{S_{2}R_{B}}+ \sum _{i=1}^{2}\lambda _{i}(t)\hat{V}^{(i)}_{int}, \end{aligned}$$

where $\hat{H}_{S}=\sum _{i=1}^{2}\hat{H}_{S_{i}}$, $\hat{H}_{R}=\sum _{l=A,B,C}\hat{H}_{R_{l}}$, and $\hat{V}^{(i)}_{int}$ is the interaction Hamiltonian of $S_{i}$ with $R_{C}$. In Eq. (3), the step function $\lambda _{i}(t)$ represents the time-dependence of the collisions between the system and the reservoir $R_{C}$, which takes the value 1 when $t\in \left[ (n-2+i)\tau , (n-1+i)\tau \right]$ with $n\ge 1$ the number of collisions, and zero otherwise. After a step of collision, the state $\rho _{S}$ of the system at time *t* evolves to $\rho _{S}^{\prime }$ at time $t+2\tau$ taking the form of

4 $$\begin{aligned} \rho _{S}^{\prime }=\textrm{tr}_{R_{C}}\rho _{SR_{C}}^{\prime }=\textrm{tr}_{R_{C}}\left\{ \hat{U}_{2}(\tau )\hat{U}_{1}(\tau )\rho _{SR_{C}} \hat{U}_{1}^{\dagger }(\tau )\hat{U}_{2}^{\dagger }(\tau )\right\} , \end{aligned}$$

in which $\hat{U}_{i}(\tau )=e^{-i\tau (\hat{H}_{S_{i}}+ \hat{H}_{R_{C}}+\hat{V}^{(i)}_{int})}$ is the unitary time evolution operator, $\rho _{SR_{C}}=\rho _{S}\otimes \rho ^{th}_{R_{C}}$ and $\rho ^{th}_{R_{C}}=e^{-\beta _{C}\hat{H}_{R_{C}}}/Z_{R_{C}}$ the initial thermal state of the reservoir $R_{C}$ at inverse temperature $\beta _{C}=1/T_{C}$ with $Z_{R_{C}}=\textrm{tr}\left\{ e^{-\beta _{C}\hat{H}_{R_{C}}}\right\}$ the partition function. We adopt $\hbar =k_{B}=1$ here and throughout the paper.

The master equation that depicts the system’s dynamics can be formulated as

5 $$\begin{aligned} \dot{\rho _{S}}=-i\left[ \hat{H_{S}},\rho _{S}\right] +\mathcal {L}_{A}\left( \rho _{S}\right) +\mathcal {L}_{B}\left( \rho _{S}\right) +\mathcal {L}_{C}\left( \rho _{S}\right) \end{aligned}$$

where $\mathcal {L}_{A}$ and $\mathcal {L}_{B}$ denote the dissipations of the subsystems $S_{1}$ and $S_{2}$ due to the coupling with thermal reservoirs $R_{A}$ and $R_{B}$, respectively, while $\mathcal {L}_{C}$ represents the dissipation of the system due to collisions with the intermediate reservoir $R_{C}$. In the above formula (5), the dissipative operators $\mathcal {L}_{A}\left( \rho _{S}\right)$ and $\mathcal {L}_{B}\left( \rho _{S}\right)$ are introduced phenomenologically, which can be expressed as

6 $$\begin{aligned} \mathcal {L}_A\left( \rho _{S}\right)= & \Gamma _A\left[ \bar{n}_{A}\left( \omega _{S_{1}}\right) +1\right] \left( \hat{\sigma }_{1}^{-}\rho _S\hat{\sigma }_{1}^{+}- \frac{1}{2}\left[ \hat{\sigma }_{1}^{+}\hat{\sigma }_{1}^{-},\rho _{\textrm{S}}\right] _{+}\right) \nonumber \\ & +\Gamma _A\bar{n}_{A}\left( \omega _{S_{1}}\right) \left( \hat{\sigma }_{1}^{+}\rho _S\hat{\sigma }_{1}^{-}- \frac{1}{2}\left[ \hat{\sigma }_{1}^{-}\hat{\sigma }_{1}^{+},\rho _{\textrm{S}}\right] _{+}\right) \end{aligned}$$

and

7 $$\begin{aligned} \mathcal {L}_B\left( \rho _{S}\right)= & \Gamma _B\left[ \bar{n}_{B}\left( \omega _{S_{2}}\right) +1\right] \left( \hat{\sigma }_{2}^{-}\rho _S\hat{\sigma }_{2}^{+}- \frac{1}{2}\left[ \hat{\sigma }_{2}^{+}\hat{\sigma }_{2}^{-},\rho _{\textrm{S}}\right] _{+}\right) \nonumber \\ & +\Gamma _B\bar{n}_{B}\left( \omega _{S_{2}}\right) \left( \hat{\sigma }_{2}^{+}\rho _S\hat{\sigma }_{2}^{-}- \frac{1}{2}\left[ \hat{\sigma }_{2}^{-}\hat{\sigma }_{2}^{+},\rho _{\textrm{S}}\right] _{+}\right) , \end{aligned}$$

respectively. In Eqs. (6) and (7), $\Gamma _A$ ($\Gamma _B$) denotes the damping rates of the reservoir $R_{A}$ ($R_B$), and $\bar{n}_{A}(\omega _{S_{1}})$ ($\bar{n}_{B}(\omega _{S_{2}})$) is the average number of photons of $R_{A}$ ($R_{B}$) at the frequency $\omega _{S_{1}}$ ($\omega _{S_{2}}$) of the subsystem $S_{1}$ ($S_2$) which takes the form

8 $$\begin{aligned} \bar{n}_{l}\left( \omega _{S_{i}}\right) =\frac{1}{\exp \left( \frac{\omega _{S_{i}}}{T_{l}}\right) -1}. \end{aligned}$$

By expanding $\hat{U}_{i}(\tau )$ to the second order of $\tau$, we obtain the dissipative operator $\mathcal {L}_{C}\left( \rho _{S}\right)$, which can be decomposed into the following form

9 $$\begin{aligned} \mathcal {L}_{C}\left( \rho _{S}\right) =\sum ^{2}_{i=1}\mathcal {L}_{i}\left( \rho _{S}\right) +\mathcal {D}_{12}\left( \rho _{S}\right) \end{aligned}$$

with

10 $$\begin{aligned} \mathcal {L}_{i}\left( \rho _{S}\right) =-\frac{1}{2}\textrm{tr}_{R_{C}}\left\{ \left[ \hat{V}^{(i)}_{int}, \left[ \hat{V}^{(i)}_{int},\rho _{S}\otimes \rho ^{th}_{R_{C}}\right] \right] \right\} \end{aligned}$$

representing the local dissipation of $S_{i}$ as if only $S_{i}$ exists and

11 $$\begin{aligned} \mathcal {D}_{12}\left( \rho _{S}\right) =-\textrm{tr}_{R_{C}}\left\{ \left[ \hat{V}^{(2)}_{int}, \left[ \hat{V}^{(1)}_{int},\rho _{S}\otimes \rho ^{th}_{R_{C}}\right] \right] \right\} \end{aligned}$$

characterizing the collective dissipation of the two subsystems due to their cascaded interactions with $R_{C}$. Here, the dissipator $\mathcal {D}_{12}$ incorporates the one-way influence between subsystems, which alternatively can also be manifested in a chiral effective Hamiltonian of the system^57^.

In the following illustrations, the interactions between the subsystems $S_{1}$ and $S_{2}$ and the reservoir $R_{C}$ are taken to be

12 $$\begin{aligned} \hat{V}_{int}^{(1)}=\frac{1}{\sqrt{\tau }}\left( J_{1}^{x} \hat{\sigma }_{S_{1}}^{x} \hat{\sigma }_{R_{C}}^x+J_{1}^{y} \hat{\sigma }_{S_{1}}^{y} \hat{\sigma }_{R_{C}}^{y}\right) , \end{aligned}$$

and

13 $$\begin{aligned} \hat{V}_{int}^{(2)}=\frac{1}{\sqrt{\tau }}\left( J_{2}^{x} \hat{\sigma }_{S_{2}}^x \hat{\sigma }_{R_{C}}^x+J_{2}^{y} \hat{\sigma }_{S_{2}}^{y} \hat{\sigma }_{R_{C}}^{y}\right) , \end{aligned}$$

respectively, in which the interactions have been scaled by the collision duration $\tau$ for the convenience of taking continuous time limit, although this is not strictly necessary. As a result, the local and non-local dissipations, given by Eqs.(10) and (11), can be updated to

14 $$\begin{aligned} \mathcal {L}_{i}\left( \rho _{S}\right)= & \left( J_{i}^{x}\right) ^{2}\left( \hat{\sigma }_{S_{i}}^{x} \rho _{S} \hat{\sigma }_{S_{i}}^{x}-\frac{1}{2}\left[ \rho _{S}, \hat{\sigma }_{S_{i}}^{x} \hat{\sigma }_{S_{i}}^{x}\right] _{+}\right) \nonumber \\ & +\left( J_{i}^{y}\right) ^{2}\left( \hat{\sigma }_{S_{i}}^{y} \rho _{S} \hat{\sigma }_{S_{i}}^{y}-\frac{1}{2}\left[ \rho _{S}, \hat{\sigma }_{S_{i}}^{y} \hat{\sigma }_{S_{i}}^{y}\right] _{+}\right) \nonumber \\ & -i J_{i}^{x} J_{i}^{y}\left\langle \hat{\sigma }_{R_{C}}^{z}\right\rangle _{\rho _{R_{C}}^{th}}\left[ \left( \hat{\sigma }_{S_{i}}^{x} \rho _{S} \hat{\sigma }_{S_{i}}^{y}-\frac{1}{2}\left[ \rho _{S}, \hat{\sigma }_{S_{i}}^{y} \hat{\sigma }_{S_{i}}^{x}\right] _{+}\right) \right. \nonumber \\ & \left. -\left( \hat{\sigma }_{S_{i}}^{y} \rho _{S} \hat{\sigma }_{S_{i}}^{x}-\frac{1}{2}\left[ \rho _{S}, \hat{\sigma }_{S_{i}}^{x} \hat{\sigma }_{S_{i}}^{y}\right] _{+}\right) \right] , \end{aligned}$$

and

15 $$\begin{aligned} \mathcal {D}_{12}\left( \rho _{S}\right)= & i\left\langle \hat{\sigma }_{R_{C}}^{z}\right\rangle _{\rho _{R_{C}}^{th}}\left\{ \left( J_{1}^{x} J_{2}^{y}\left[ \hat{\sigma }_{S_{2}}^{y}, \rho _{S} \hat{\sigma }_{S_{1}}^{x}\right] +J_{1}^{y} J_{2}^{x}\left[ \hat{\sigma }_{S_{1}}^{y} \rho _{S}, \hat{\sigma }_{S_{2}}^{x}\right] \right) \right. \nonumber \\ & \left. -\left( J_{1}^{y} J_{2}^{x}\left[ \hat{\sigma }_{S_{2}}^{x}, \rho _{S} \hat{\sigma }_{S_{1}}^{y}\right] +J_{1}^{x} J_{2}^{y}\left[ \hat{\sigma }_{S_{1}}^{x} \rho _{S}, \hat{\sigma }_{S_{2}}^{y}\right] \right) \right\} \nonumber \\ & +J_{1}^{x} J_{2}^{x}\left[ \hat{\sigma }_{S_{2}}^{x},\left[ \rho _{S}, \hat{\sigma }_{S_{1}}^{x}\right] \right] +J_{1}^{y} J_{2}^{y}\left[ \hat{\sigma }_{S_{2}}^{y},\left[ \rho _{S}, \hat{\sigma }_{S_{1}}^{y}\right] \right] , \end{aligned}$$

with $$\left\langle \cdot \right\rangle _{\rho }\equiv \textrm{tr}[\cdot \rho ]$$.

To compare the cascaded interaction with simultaneous interaction between the two subsystems and the reservoir $R_{C}$, we also present the master equation for the latter case. For the simultaneous interaction, the structure of local dissipation remains the same as that given in Eq. (10), while the non-local dissipation can be expressed as

16 $$\begin{aligned} \mathcal {D}_{12}^{\text{ sim }}\left( \rho _{S}\right)= & -\frac{1}{2} \operatorname {tr}_{R_{C}} \left[ \hat{V}^{(1)}_{int},\left[ \hat{V}^{(2)}_{int}, \rho _{S} \otimes \rho _{R_{C}}^{th}\right] \right] \nonumber \\ & -\frac{1}{2}\operatorname {tr}_{R_{C}}\left[ \hat{V}^{(2)}_{int}, \left[ \hat{V}^{(1)}_{int}, \rho _{S} \otimes \rho _{R_{C}}^{th}\right] \right] . \end{aligned}$$

The non-local dissipation term of the simultaneous collision is more “symmetric” in comparison to the situation of cascaded collision. Taking into account the specific forms of interactions as given in Eqs. (12) and (13), the non-local dissipation can be expressed as

17 $$\begin{aligned} \mathcal {D}_{12}^{\text{ sim } }\left( \rho _{S}\right)= & \frac{i}{2}\left\langle \hat{\sigma }_{R_{C}}^{z}\right\rangle _{\rho _{R_{C}}^{th}}\left( J_1^x J_2^y\left( \left[ \hat{\sigma }_{S_2}^y, \rho _S \hat{\sigma }_{S_1}^x\right] +\left[ \hat{\sigma }_{S_2}^y \rho _{S}, \hat{\sigma }_{S_1}^x\right] \right) \right. \nonumber \\ & \left. +J_1^y J_2^x\left( \left[ \hat{\sigma }_{S_1}^y \rho _{S}, \hat{\sigma }_{S_2}^x\right] +\left[ \hat{\sigma }_{S_1}^y, \rho _{S} \hat{\sigma }_{S_2}^x\right] \right) \right) \nonumber \\ & -\frac{i}{2}\left\langle \hat{\sigma }_{R_{C}}^{z}\right\rangle _{\rho _{R_{C}}^{th}}\left( J_1^y J_2^x\left( \left[ \hat{\sigma }_{S_2}^x, \rho _{S} \hat{\sigma }_{S_1}^y\right] +\left[ \hat{\sigma }_{S_2}^x \rho _{S}, \hat{\sigma }_{S_1}^y\right] \right) \right. \nonumber \\ & \left. +J_1^x J_2^y\left( \left[ \hat{\sigma }_{S_1}^x \rho _{S}, \hat{\sigma }_{S_2}^y\right] +\left[ \hat{\sigma }_{S_1}^x, \rho _S \hat{\sigma }_{S_2}^y\right] \right) \right) \nonumber \\ & +\frac{J_1^x J_2^x}{2}\left( \left[ \hat{\sigma }_{S_1}^x\left[ \rho _{S}, \hat{\sigma }_{S_2}^x\right] \right] +\left[ \hat{\sigma }_{S_2}^x\left[ \rho _{S}, \hat{\sigma }_{S_1}^x\right] \right] \right) \nonumber \\ & +\frac{J_1^y J_2^y}{2}\left( \left[ \hat{\sigma }_{S_1}^y\left[ \rho _{S}, \hat{\sigma }_{S_2}^y\right] \right] +\left[ \hat{\sigma }_{S_2}^y\left[ \rho _{S}, \hat{\sigma }_{S_1}^y\right] \right] \right) . \end{aligned}$$

##  Thermodynamic quantities

The heat currents from the reservoirs $R_{A}$ and $R_{B}$ to the system can be defined as the energy change of the system due to the dissipations of the reservoirs, which are formulated as $J_{A} =\operatorname {Tr}\left\{ \mathcal {L}_A\left[ \rho _{S}\right] \hat{H}_{S_1}\right\}$ and $J_{B} =\operatorname {Tr}\left\{ \mathcal {L}_B\left[ \rho _{S}\right] \hat{H}_{S_2}\right\}$, respectively. For the dissipations given in Eqs. (6) and (7), we can obtain the explicit forms of heat currents as

18 $$\begin{aligned} J_{A} =\Gamma _A \omega _{S_{1}}\left( n_A\left\langle \hat{\sigma }_{S_{1}}^{-} \hat{\sigma }_{S_{1}}^{+}\right\rangle _{\rho _S} -\left( n_A+1\right) \left\langle \hat{\sigma }_{S_{1}}^{+} \hat{\sigma }_{S_{1}}^{-}\right\rangle _{\rho _S}\right) , \end{aligned}$$

and

19 $$\begin{aligned} J_{B} =\Gamma _B \omega _{S_{2}}\left( n_B\left\langle \hat{\sigma }_{S_{2}}^{-} \hat{\sigma }_{S_{2}}^{+}\right\rangle _{\rho _S}-\left( n_B+1\right) \left\langle \hat{\sigma }_{S_{2}}^{+} \hat{\sigma }_{S_{2}}^{-} \right\rangle _{\rho _S}\right) , \end{aligned}$$

in which $n_A\equiv \bar{n}_A(\omega _{S_1})$ and $n_B\equiv \bar{n}_B(\omega _{S_2})$.

The heat transferred from the middle reservoir $R_{C}$ to the system can be quantified by the energy variations of $R_{C}$ during a collision as

20 $$\begin{aligned} \Delta J_{C}=-\operatorname {tr}\left\{ \hat{H}_{R_{C}}\left( \rho _{SR_{C}}^{\prime }-\rho _{SR_{C}}\right) \right\} . \end{aligned}$$

We note that due to the collective interaction between $R_{C}$ and the two subsystems $S_{1}$ and $S_{2}$, the heat can be decomposed into the local component $\Delta J_{C}^{(i)}$ associated with $S_{i}$ and nonlocal one $\Delta J_{C}^{(12)}$ related to the nonlocal dissipation of $R_{C}$, that is, $\Delta J_{C}=\sum _{i=1}^2 \Delta J_{C}^{(i)}+\Delta J_{C}^{(12)}$. By taking the continuous time limit, we get the heat current as

21 $$\begin{aligned} J_{C}=-\lim _{\tau \rightarrow 0} \frac{\Delta J_{C}}{\tau } =\sum _{i=1}^2 J_{C}^{(i)}+J_{C}^{(12)}. \end{aligned}$$

According to the map given in Eq. (4), the local and nonlocal components of heat current $J_{C}$ can be derived as

22 $$\begin{aligned} J_{C}^{(i)}=\frac{1}{2} \operatorname {tr}\left\{ \left[ \hat{V}_{int}^{(i)}, \left[ \hat{H}_{R_{C}}, \hat{V}_{int}^{(i)}\right] \right] \rho _{SR_{C}}\right\} \end{aligned}$$

and

23 $$\begin{aligned} J_{C}^{(12)}=\operatorname {tr}\left\{ \left[ \hat{V}_{int}^{(1)}, \left[ \hat{H}_{R_{C}}, \hat{V}_{int}^{(2)}\right] \right] \rho _{SR_{C}}\right\} , \end{aligned}$$

respectively. For the specific interaction form shown in Eqs. (12) and (13), $J_{C}^{(i)}$ and $J_{C}^{(12)}$ can be further expressed as

24 $$\begin{aligned} J_{C}^{(i)}=-\omega _{C}\left[ 2 J_i^x J_i^y\left\langle \sigma _{S_i}^z\right\rangle _{\rho _S} -\left( (J_i^x)^2+(J_i^y)^2\right) \left\langle \sigma _{R_{C}}^z\right\rangle _{\rho _{R_{{C}}}^{th}}\right] , \end{aligned}$$

and

25 $$\begin{aligned} J_{C}^{(12)}=2 \omega _{C}\left\langle \sigma _{R_{C}}^z\right\rangle _{\rho _{R_{C}}^{th}}\left( J_1^x J_2^x\left\langle \sigma _{S_1}^x \sigma _{S_2}^x\right\rangle _{\rho _S}+J_1^y J_2^y\left\langle \sigma _{S_1}^y \sigma _{S_2}^y\right\rangle _{\rho _S}\right) , \end{aligned}$$

respectively.

Work in quantum thermodynamics is commonly defined as the alteration in energy of the total system resulting from time-variation of the Hamiltonian. Within the framework of collision model, the sequential couplings and decouplings of the system with the reservoir $R_{C}$ give rise to the temporal dependence of the interaction Hamiltonian, as given in Eq.(3). Consequently, the energetic cost required to maintain these consecutive collisions is supplied in the form of work. In a round of collision from *t* to $t+2 \tau$, the work performed on the system is formulated as

26 $$\begin{aligned} \Delta W=\int _t^{t+2 \tau }\left\langle \frac{\partial \hat{H}_{tot}(s)}{\partial s}\right\rangle _{\rho _{SR_{{C}}}} ds. \end{aligned}$$

From the Hamiltonian $\hat{H}_{tot}(s)$, Eq. (3), we can see that only the interactions of the two subsystems with the reservoir $R_{C}$ are time-dependent. After taking the continuous time limit, we can obtain the work current $\dot{W}=\lim _{\tau \rightarrow 0} \Delta W/\tau$. An integration over Eq. (26) yields

27 $$\begin{aligned} \dot{W}=\sum _{i=1}^2 \dot{W}_i+\dot{W}_{12}, \end{aligned}$$

with

28 $$\begin{aligned} \dot{W}_i=\frac{1}{2} \operatorname {tr}\left\{ \left[ \hat{V}_{i n t}^{(i)},\left[ \hat{H}_{S_i}+\hat{H}_{R_{C}}, \hat{V}_{i n t}^{(i)}\right] \right] \rho _{SR_{C}}\right\} , \end{aligned}$$

and

29 $$\begin{aligned} \dot{W}_{12}=\operatorname {tr}\left\{ \left[ \hat{V}_{i n t}^{(1)},\left[ \hat{H}_{S_2}+\hat{H}_{R_{C}}, \hat{V}_{i n t}^{(2)}\right] \right] \rho _{SR_{C}}\right\} , \end{aligned}$$

being the local and nonlocal components of the work current. Eq. (28) indicates that if $[\hat{H}_{S_i}+\hat{H}_{R_{C}},$ $\hat{V}_{i n t}^{(i)}]=0$, namely, the interaction between the subsystem $S_{i}$ and the reservoir $R_{C}$ conserves energy, the local work current $\dot{W}_i$ vanish. Moreover, when the local work current $\dot{W}_2$ is zero, the non-local work current $\dot{W}_{12}$ must also be zero. However, as can be seen from the subsequent formula (31), $\dot{W}_{12}$ also depend on the correlations established between $S_1$ and $S_2$. Even if $W_2$ is not zero, the absence of correlations between $S_1$ and $S_2$ in terms of $\left\langle \sigma _{S_1}^x \sigma _{S_2}^x\right\rangle _{\rho _S}$ and $\left\langle \sigma _{S_1}^y \sigma _{S_2}^y\right\rangle _{\rho _S}$ will also lead to the disappearance of $\dot{W}_{12}$. For the specific interactions given in Eqs. (12) and (13), the local work current reads

30 $$\begin{aligned} \dot{W}_i= & \omega _{S_i}\left( 2 J_i^x J_i^y\left\langle \sigma _{R_{C}}^z\right\rangle _{\rho _{R_{C}}^{th}} -\left( (J_i^x)^2+(J_i^y)^2\right) \left\langle \sigma _{S_i}^z\right\rangle _{\rho _S}\right) \nonumber \\ & +\omega _{C}\left( 2 J_i^x J_i^y\left\langle \sigma _{S_i}^z\right\rangle _{\rho _S} -\left( (J_i^x)^2+(J_i^y)^2\right) \left\langle \sigma _{R_{C}}^z\right\rangle _{\rho _{R_{C}}^{th}}\right) , \end{aligned}$$

while the nonlocal component can be expressed as

31 $$\begin{aligned} \dot{W}_{12}= & 2 J_1^x\left( \omega _{S_2} J_2^y-\omega _{C} J_2^x\right) \left\langle \sigma _{S_1}^x \sigma _{S_2}^x\right\rangle _{\rho _S}\left\langle \sigma _{R_{C}}^z\right\rangle _{\rho _{R_{C}}^{th}}\nonumber \\ & +J_1^y\left( \omega _{S_2} J_2^x-\omega _{C} J_2^y\right) \left\langle \sigma _{S_1}^y \sigma _{S_2}^y\right\rangle _{\rho _S}\left\langle \sigma _{R_{C}}^z\right\rangle _{\rho _{R_{C}}^{th}}. \end{aligned}$$

Here, we also derive the work current regarding the simultaneous interactions between the subsystems and reservoir $R_{C}$. Since the local component of the work current for the simultaneous interactions is the same as that given in Eqs. (28) and (30) for the cascaded interactions, we only present its general nonlocal component as

32 $$\begin{aligned} \dot{W}_{12}^{\text{ sim } }= & \frac{1}{2} \operatorname {tr}\left\{ \left[ \hat{V}_{\text{ int } }^{(1)},\left[ \hat{H}_{S_2}+\hat{H}_{R_{C}}, \hat{V}_{\text{ int } }^{(2)}\right] \right] \rho _{SR_{C}}\right\} \nonumber \\ & +\frac{1}{2} \operatorname {tr}\left\{ \left[ \hat{V}_{\text{ int } }^{(2)},\left[ \hat{H}_{S_1}+\hat{H}_{R_{C}}, \hat{V}_{\text{ int } }^{(1)}\right] \right] \rho _{SR_{C}}\right\} , \end{aligned}$$

and the specific form as

33 $$\begin{aligned} \dot{W}_{12}^{\text{ sim } }= & J_1^x\left( \omega _{S_2} J_2^y-\omega _{C} J_2^x\right) \left\langle \sigma _{S_1}^x \sigma _{S_2}^x\right\rangle _{\rho _S}\left\langle \sigma _{R_{C}}^z\right\rangle _{\rho _{R_{C}}^{th}}\nonumber \\ & +J_1^y\left( \omega _{S_2} J_2^x-\omega _{C} J_2^y\right) \left\langle \sigma _{S_1}^y \sigma _{S_2}^y\right\rangle _{ \rho _S}\left\langle \sigma _{R_{C}}^z\right\rangle _{\rho _{R_{C}}^{th}} \nonumber \\ & +J_2^x\left( \omega _{S_1} J_1^y-\omega _{C} J_1^x\right) \left\langle \sigma _{S_2}^x \sigma _{S_1}^x\right\rangle _{\rho _S}\left\langle \sigma _{R_{C}}^z\right\rangle _{\rho _{R_{C}}^{th}} \nonumber \\ & +J_2^y\left( \omega _{S_{1}} J_1^x-\omega _{C} J_1^y\right) \left\langle \sigma _{S_2}^y \sigma _{S_1}^y\right\rangle _{\rho _S}\left\langle \sigma _{R_{C}}^z\right\rangle _{\rho _{R_{C}}^{th}}. \end{aligned}$$

**Fig. 2:**
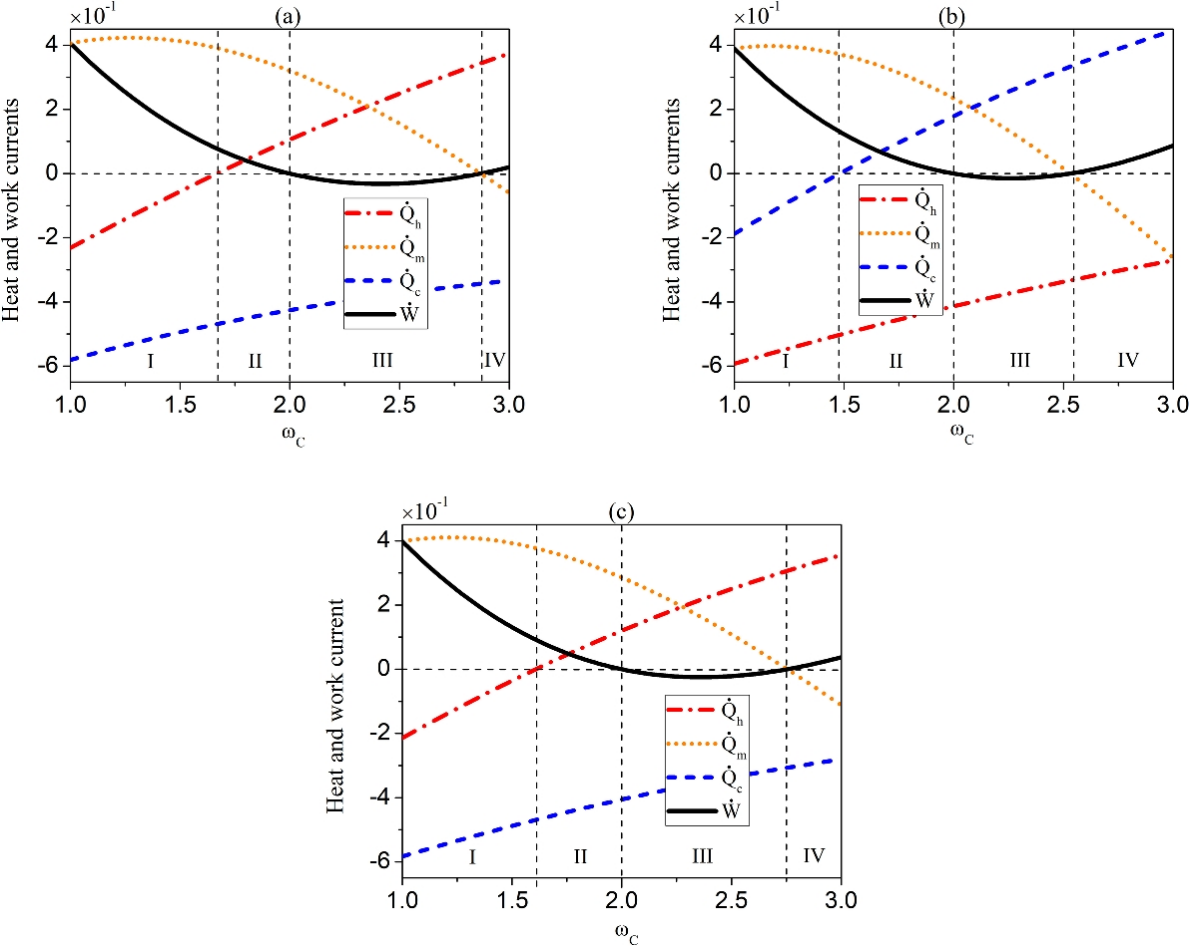
(Color online) The currents of heat and work as a function of the frequency $\omega _{{C}}$ of ancilla of the reservoir $R_{C}$ for the symmetric case. The cascaded interactions between subsystems $S_{1}$ and $S_{2}$ and $R_{C}$ for $T_{A}=2.4\omega _{C}>T_{C}=2\omega _{C}>T_{B}=0.5\omega _{{C}}$ (**a**) and $T_{A}=0.5\omega _{{C}}<T_{C}=2\omega _{{C}}<T_{B}=2.4\omega _{{C}}$ (**b**). The simultaneous interactions between $S_{1}$ and $S_{2}$ and $R_{C}$ (**c**). The other parameters are chosen as $\omega _{S_1}=\omega _{S_2}=2 \omega _{C}, J=\omega _{{C}},\Gamma _A=\Gamma _B=1$.

## Quantum thermal machines

After properly defining thermodynamic quantities, we are ready to study the functions that our setup can achieve as QTMs, as well as their performance in different scenarios. In the following, we mainly examine two configurations: one is the symmetric case where two subsystems $S_{1}$ and $S_{2}$ are resonant, and the other is the asymmetric case with detuning between them. We unveil different functions exhibited by the system relying on variations of the frequency of ancillas in the reservoir $R_C$.

**Table 1:** Table of operating regimes of the machine for the symmetric case with $\omega _{S_1}=\omega _{S_2}$.

Label	Description	$\dot{Q}_{c}$	$\dot{Q}_{m}$	$\dot{Q}_{h}$	$\dot{W}$
I	$T_{C}$-power driven refrigerator	$<0$	$>0$	$<0$	$>0$
II	Dual-source accelerator	$<0$	$>0$	$>0$	$>0$
III	Dual-source engine	$<0$	$>0$	$>0$	$<0$
IV	Dual-sink accelerator	$<0$	$<0$	$>0$	$>0$

The first column shows the labels of different regimes; the second column describes the functions and features of the machine; the remaining columns give the signs of the heat and work currents.

### Symmetric case

In order to obtain explicit operating regimes for the system, we first consider a specific situation, namely, $\omega _{S_1}=\omega _{S_2}$, $J_1^x=J_1^y=J_2^x=J_2^y=J$. In this case, we find the system can achieve four different functions within certain ranges of the frequency $\omega _{C}$ of the reservoir $R_{C}$, as shown in the Table 1. As the temperatures of the three reservoirs are not determined in prior, we have used $\dot{Q}_{h}$ and $\dot{Q}_{c}$ to represent the heat currents for reservoirs with the highest and the coldest temperatures, and $\dot{Q}_{m}$ for the reservoir with middle temperature. In the following, we shall consider two settings of the temperatures, namely, $T_{A}>T_{C}>T_{B}$ and $T_{A}<T_{C}<T_{B}$, with the temperature of reservoir $R_{C}$ always being the intermediate one.

We first consider the case of $T_{A}>T_{C}>T_{B}$, for which $\dot{Q}_{h}\equiv J_{A}$, $\dot{Q}_{c}\equiv J_{B}$, and $\dot{Q}_{m}\equiv J_{C}$. For the operating regime of Type I, namely, $\dot{W}>0$, $\dot{Q}_{m}>0$ and $\dot{Q}_{h}<0$, the external agent performs work on the system, driving heat flow from the reservoir $R_{C}$ with middle temperature to $R_{A}$ with the highest temperature. Hence, the machine of Type I realizes the cooling of reservoir $R_{C}$, which is thus called the $T_{C}$-power driven-refrigerator. In the regime of Type II, apart from the external work on the system with $\dot{W}>0$, the heat is transferred from the reservoirs $R_{A}$ and $R_{C}$ with relatively high temperatures to the reservoir $R_{B}$ with relatively low temperature with $\dot{Q}_{m}>0$, $\dot{Q}_{h}>0$ and $\dot{Q}_{c}<0$. Therefore, the machine of Type II operates as the dual-source accelerator (oven)^65^ by acquiring external work and transporting heat from the dual sources $R_{A}$ and $R_{C}$ to $R_{B}$. The machine of Type III with $\dot{W}<0$, $\dot{Q}_{m}>0$, $\dot{Q}_{h}>0$ and $\dot{Q}_{c}<0$ obviously implements the dual-source engine, which perform work to an external agent by transferring heat from the reservoirs $R_{C}$ and $R_{A}$ to $R_{B}$. The Type IV of QTMs is also an accelerator, but unlike the Type II, it absorbs heat from the high-temperature reservoir $R_{A}$ and dumps the heat into two relatively cooler reservoirs $R_{B}$ and $R_{C}$, which is thus called dual-sink accelerator. In Fig. 2a, we plot the work current $\dot{W}$ and the three heat currents $\dot{Q}_{m}$, $\dot{Q}_{h}$ and $\dot{Q}_{c}$ with respect to the frequency $\omega _{C}$ of the ancilla of reservoir $R_{C}$. We have labeled the regions for appearances of different operating regimes according to the Table 1.

By reversing the temperatures of $R_A$ and $R_B$ to be $T_{A}<T_{C}<T_{B}$, we can still obtain the same four types of operating regimes as the case of $T_{A}>T_{C}>T_{B}$. However, the ranges in which these operating regimes appear are altered, as shown in Fig. 2b. This reflects the asymmetric characteristic of the cascaded interactions occurring between subsystems $S_{1}$ and $S_{2}$ with $R_{C}$.

In order to compare the cascaded interaction with simultaneous interaction, we illustrate the heat and work currents under the latter case in Fig. 2c. We can see that the simultaneous interaction does not change the types of QTMs compared to the cascaded interaction, but shifts the intervals of their occurrences. Moreover, if the temperatures of the reservoirs $R_A$ and $R_B$ are reversed, there will be no influence on the results due to the symmetry character of the simultaneous interaction.

Next, we demonstrate the influences of different configurations, namely, the cascaded interaction with $T_{A}>T_{C}>T_{B}$ and $T_{A}<T_{C}<T_{B}$ as well as the simultaneous interaction, on the performance of QTMs. Here, we focus on the dual-source engine, which appears in the common interval $\omega _{C} \in (2.1,2.5)$ for all these three situations. We use the efficiency to assess the performance of quantum engine, which can be defined as

34 $$\begin{aligned} \eta =\frac{\left| \dot{W}\right| }{\dot{Q}_{h}+\dot{Q}_{m}}. \end{aligned}$$

**Fig. 3:**
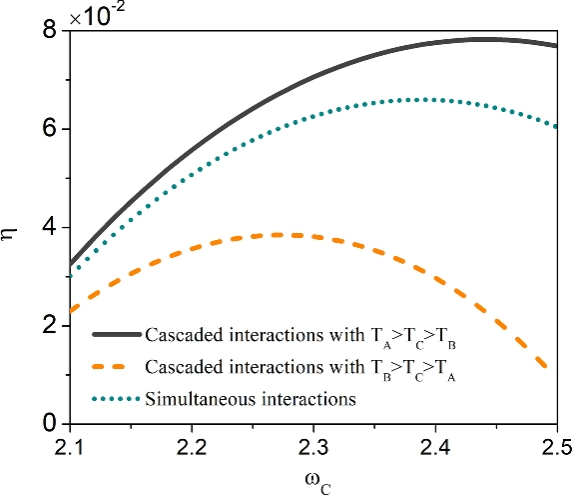
(Color online) The efficiency of dual-source engine under three situations, i.e., the cascaded interactions between the two subsystems and the reservoir $R_{C}$ with $T_{A}>T_{B}>T_{C}$ and $T_{A}<T_{B}<T_{C}$, and the simultaneous interactions. The other parameters are the same as that used in Fig. 2.

We exhibit in Fig. 3 the efficiency $\eta$ of dual-source engine with respect to $\omega _{C}$ for the three situations, i.e., the cascaded interaction with $T_{A}>T_{C}>T_{B}$ and $T_{A}<T_{C}<T_{B}$ and the simultaneous interaction. We observe that the cascaded interaction for the temperature setting $T_{A}>T_{C}>T_{B}$ can lead to the largest efficiency, while the configuration $T_{A}<T_{C}<T_{B}$ causes the lowest efficiency. The efficiency of simultaneous interaction is always in between these two cases.

### Asymmetric case

**Table 2:** Table of operating regimes of the machine for asymmetric case with $\omega _{S_1}\ne \omega _{S_2}$.

Label	Description	$\dot{Q}_{c}$	$\dot{Q}_{m}$	$\dot{Q}_{h}$	$\dot{W}$
I	$T_{C}$-power driven refrigerator	$<0$	$>0$	$<0$	$>0$
III	Dual-source engine	$<0$	$>0$	$>0$	$<0$
V	$T_{C}$-heat-driven refrigerator with work production	$<0$	$>0$	$<0$	$<0$
VI	Dual-sink engine	$<0$	$<0$	$>0$	$<0$

The first column shows the labels of different regimes; the second column describes the functions and characters of the machine; the remaining columns give the signs of heat and work currents.

**Fig. 4:**
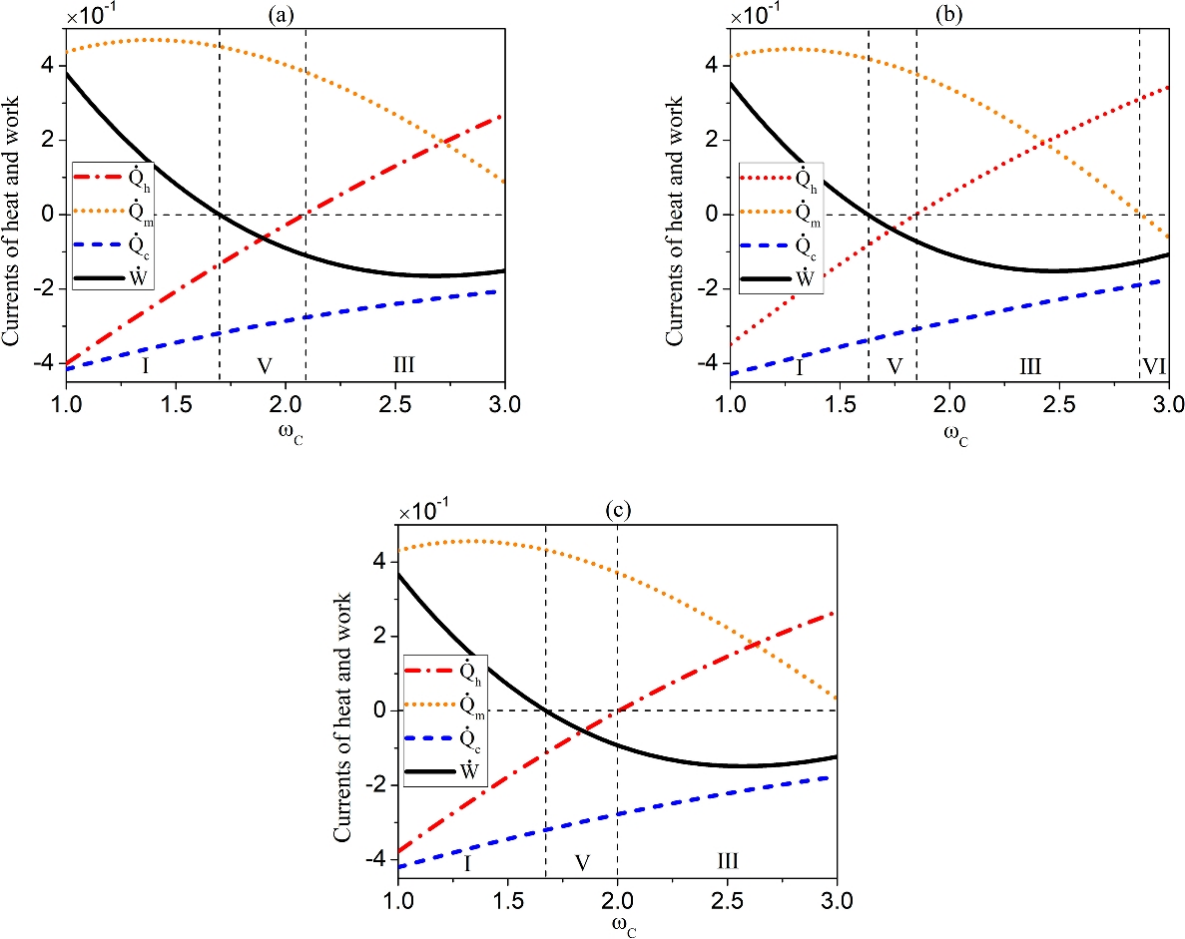
(Color online) The currents of heat and work as a function of the frequency $\omega _{C}$ of ancilla of the reservoir $R_{C}$ for the asymmetric case. The cascaded interactions between subsystems $S_{1}$ and $S_{2}$ and $R_{C}$ for $T_{A}=2.4\omega _{C}>T_{C}=2\omega _{C}>T_{B}=0.5\omega _{C}$ and $\omega _{S_1}=2.5\omega _{C}$, $\omega _{S_2}=1.5\omega _{C}$ (**a**), and $T_{A}=0.5\omega _{C}<T_{C}=2\omega _{C}<T_{B}=2.4\omega _{C}$ and $\omega _{S_1}=1.5\omega _{C}$, $\omega _{S_2}=2.5\omega _{C}$ (**b**). The simultaneous interactions between $S_{1}$ and $S_{2}$ and $R_{C}$ (**c**). The other parameter is the same as that used in Fig. 2.

**Fig. 5:**
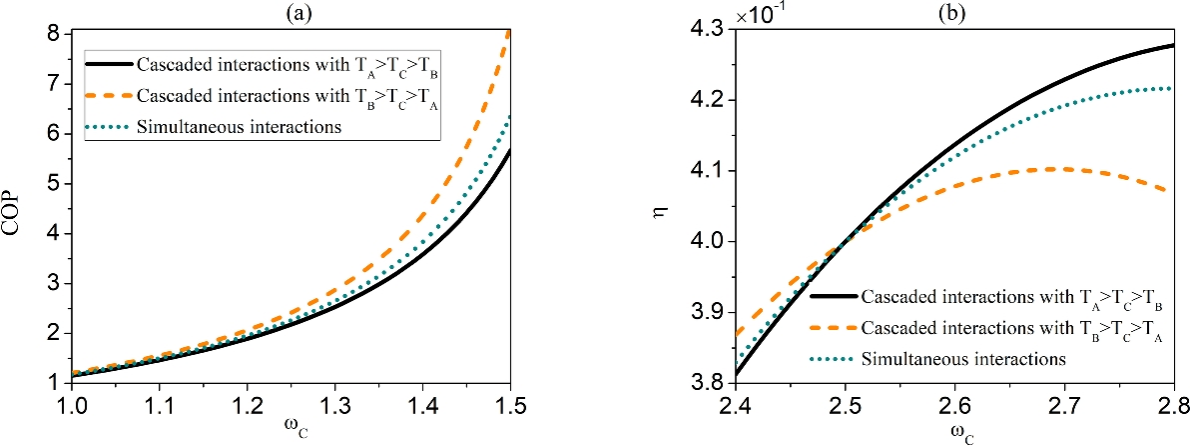
(Color online) The COP of the $T_{C}$-power driven refrigerator (**a**) and the efficiency $\eta$ of the dual-sink engine versus $\omega _{C}$. The parameters are the same as that used in Fig.4.

In the following, we further consider the operating modes of our setup under the asymmetric situation where the two subsystems exhibit different frequencies, namely, $\omega _{S_1}\ne \omega _{S_2}$. For the interactions between the subsystems and the reservoir $R_{C}$, we still assume $J_1^x=J_1^y=J_2^x=J_2^y=J$. The results are presented in Table 2, which shows that apart from the Types I and III that have been achieved in the symmetric case, as shown in Table 1, two new types of thermal machines, i.e., Type V and Type VI, arise. For the QTM of Type V, called $T_{C}$-heat-driven refrigerator with work production, the heat current is transferred from the reservoir with an intermediate temperature to the reservoir with the highest temperature, functioning as a refrigerator, and at the same time the work is produced instead of being consumed. In the case of Type VI, the system perform work on an external agent by absorbing heat from the hot reservoir and releasing it to two other reservoirs, which is thus called dual-sink engine.

In Fig. 4a, we illustrate the operating regimes of the machines by plotting the work and heat currents against $\omega _{C}$ for the cascaded interactions with $T_{A}>T_{C}>T_{B}$, for which there appear three functions of the machines. In Fig. 4b, we exhibit the results by reversing the temperatures of $R_{A}$ and $R_{B}$ to be $T_{A}<T_{C}<T_{B}$ and meanwhile exchanging the positions of the two subsystems, which shows that all the four operating regimes listed in Table 2 can be observed. Therefore, the system manifests different functions in these two scenarios embodying the unidirectional feature of the cascaded interaction. Fig. 4c displays the case of simultaneous interaction of $S_{1}$ and $S_{2}$ with $R_{C}$, from which we can see that three types of machines arise, similar to that exhibited in Fig. 4a, but with different intervals.

Next, we compare the efficiency of the machines under different configurations by focusing on Type I (i.e., $T_{C}$-power driven refrigerator) and Type III (i.e., dual-source engine) of the machines. The coefficient of performance (COP) of a refrigerator is defined as

35 $$\begin{aligned} COP=\frac{\dot{Q}_{c}}{\dot{W}}, \end{aligned}$$

while the efficiency of the dual-sink engine is still quantified by the formula Eq. (34). In Fig. 5a and b, we show the COP of $T_{C}$-power driven refrigerator and the efficiency of dual-source engine as a function of $\omega _{C}$ for different situations. From Fig. 5a, we observe that the COP of the refrigerator for cascaded interaction entailing $T_{B}>T_{C}>T_{A}$ achieves the highest efficiency, while that with temperature $T_{A}>T_{C}>T_{B}$ has the lowest efficiency. The COP of refrigerator under simultaneous interaction falls between the two. From Fig. 5b, it can be seen that the efficiency of the engine relies on $\omega _{C}$: for relatively small values of $\omega _{C}$, the cascaded interaction with the temperature setting $T_{B}>T_{C}>T_{A}$ results in the highest efficiency, while for relatively large $\omega _{C}$, the cascaded interaction with $T_{A}>T_{C}>T_{B}$ leads to the highest efficiency. The efficiency under the simultaneous interaction always falls between the two.

## Conclusion

In conclusion, we have studied the impacts of the sequence of system-environment interactions on the operating regimes and performance of QTMs. The working substance of our setup consists of two subsystems, $S_1$ and $S_2$, coupled to their local reservoirs $R_A$ and $R_B$, respectively, and meanwhile to the third reservoir $R_C$ through sequential interactions. For comparison, we have also considered the situation that the two subsystems interact simultaneously with $R_C$. Our findings indicate that the unidirectionality resulting from the sequential interaction between the subsystems and the reservoir $R_C$ can influence both the functions and performance of QTMs. In the symmetric scenario where the two subsystems are resonant, although exchanging the temperatures of two local reservoirs $R_{A}$ and $R_{B}$ does not alter the functions of QTMs, such a swap significantly changes their efficiency: in one case, the efficiency surpasses that achieved when the system interacts simultaneously with the common reservoir $R_C$, whereas in another case, it falls below this level. In the antisymmetric scenario where the two subsystems are detuning, the exchange of $R_{A}$ and $R_{B}$ influences both the types of QTMs and their performance. Our results reveal the influence of the sequence of interactions between subsystems and the reservoir on the function and performance of QTMs, providing a potential means for their design and performance enhancement.

## Data Availability

The datasets used and/or analyzed during the current study are available from the corresponding author on reasonable request.

## References

[1] Nielsen, M. & Chuang, I. *Quantum Computation and Quantum Information* (Cambridge University Press, Cambridge, 2000).

[2] Gemma, G., Michel, M. & Mahler, G. *Quantum Thermodynamics* (Springer, Berlin, 2004).

[3] Deffner, S. & Campbell, S. *Quantum Thermodynamics: An introduction to the thermodynamics of quantum information*. 2053–2571 (Morgan and Claypool Publishers, 2019). 10.1088/2053-2571/ab21c6.

[4] Kosloff, R. Quantum thermodynamics: A dynamical viewpoint. *Entropy* **15**, 2100. 10.3390/e15062100 (2013).

[5] Vinjanampathy, S. & Anders, J. Quantum thermodynamics. *Contemp. Phys.* **57**, 545. 10.1080/00107514.2016.1201896 (2016).

[6] Goold, J., Huber, M., Riera, A., del Rio, L. & Skrzypczyk, P. The role of quantum information in thermodynamics-a topical review. *J. Phys. A: Math. Theor.* **49**, 143001. 10.1088/1751-8113/49/14/143001 (2016).

[7] Millen, J. & Xuereb, A. Perspective on quantum thermodynamics. *New J. Phys.* **18**, 011002. 10.1088/1367-2630/18/1/011002 (2016).

[8] F. Binder, Correa, C. G. J. A. L. A. & Adesso, G. E. *Thermodynamics in the Quantum Regime. Fundamental Aspects and New Directions* (Springer, Switzerland, 2018).

[9] Cangemi, L. M., Bhadra, C. & Levy, A. Quantum engines and refrigerators. *Phys. Rep.* **1087**, 1 (2024).

[10] Mitchison, M. T. Quantum thermal absorption machines: Refrigerators, engines and clocks. *Contemp. Phys.* **60**, 164 (2019).

[11] Scovil, H. E. D. & Schulz-DuBois, E. O. Three-level masers as heat engines. *Phys. Rev. Lett.* **2**, 262. 10.1103/PhysRevLett.2.262 (1959).

[12] Quan, H. T., Liu, Y.-x, Sun, C. P. & Nori, F. Quantum thermodynamic cycles and quantum heat engines. *Phys. Rev. E* **76**, 031105. 10.1103/PhysRevE.76.031105 (2007). 10.1103/PhysRevE.76.031105 17930197

[13] Kaneyasu, M. & Hasegawa, Y. Quantum otto cycle under strong coupling. *Phys. Rev. E* **107**, 044127. 10.1103/PhysRevE.107.044127 (2023). 37198760 10.1103/PhysRevE.107.044127

[14] Mehta, V. & Johal, R. S. Quantum otto engine with exchange coupling in the presence of level degeneracy. *Phys. Rev. E* **96**, 032110. 10.1103/PhysRevE.96.032110 (2017). 29346897 10.1103/PhysRevE.96.032110

[15] Purkait, C. & Biswas, A. Measurement-based quantum otto engine with a two-spin system coupled by anisotropic interaction: Enhanced efficiency at finite times. *Phys. Rev. E* **107**, 054110. 10.1103/PhysRevE.107.054110 (2023). 37329072 10.1103/PhysRevE.107.054110

[16] Ishizaki, M., Hatano, N. & Tajima, H. Switching the function of the quantum otto cycle in non-markovian dynamics: Heat engine, heater, and heat pump. *Phys. Rev. Res.* **5**, 023066. 10.1103/PhysRevResearch.5.023066 (2023).

[17] Cherubim, C., de Oliveira, T. R. & Jonathan, D. Nonadiabatic coupled-qubit otto cycle with bidirectional operation and efficiency gains. *Phys. Rev. E* **105**, 044120. 10.1103/PhysRevE.105.044120 (2022). 35590646 10.1103/PhysRevE.105.044120

[18] Son, J., Talkner, P. & Thingna, J. Monitoring quantum otto engines. *PRX Quantum* **2**, 040328. 10.1103/PRXQuantum.2.040328 (2021).

[19] Singh, V. & Müstecaplıoğlu, O. E. Performance bounds of nonadiabatic quantum harmonic otto engine and refrigerator under a squeezed thermal reservoir. *Phys. Rev. E* **102**, 062123. 10.1103/PhysRevE.102.062123 (2020). 33466082 10.1103/PhysRevE.102.062123

[20] Chen, J.-F., Sun, C.-P. & Dong, H. Achieve higher efficiency at maximum power with finite-time quantum otto cycle. *Phys. Rev. E* **100**, 062140. 10.1103/PhysRevE.100.062140 (2019). 31962481 10.1103/PhysRevE.100.062140

[21] Leggio, B. & Antezza, M. Otto engine beyond its standard quantum limit. *Phys. Rev. E* **93**, 022122. 10.1103/PhysRevE.93.022122 (2016). 26986303 10.1103/PhysRevE.93.022122

[22] Huang, R., Xia, Y.-J. & Man, Z.-X. Manipulation and enhancement of the performance of otto cycle in the presence of nonthermal reservoirs. *New J. Phys.* **26**, 033052. 10.1088/1367-2630/ad3573 (2024).

[23] Molitor, O. A. D. & Landi, G. T. Stroboscopic two-stroke quantum heat engines. *Phys. Rev. A* **102**, 042217. 10.1103/PhysRevA.102.042217 (2020).

[24] Melo, F. V. et al. Implementation of a two-stroke quantum heat engine with a collisional model. *Phys. Rev. A* **106**, 032410. 10.1103/PhysRevA.106.032410 (2022).

[25] Linden, N., Popescu, S. & Skrzypczyk, P. How small can thermal machines be? The smallest possible refrigerator. *Phys. Rev. Lett.* **105**, 130401. 10.1103/PhysRevLett.105.130401 (2010). 21230755 10.1103/PhysRevLett.105.130401

[26] Müller, M. P. Correlating thermal machines and the second law at the nanoscale. *Phys. Rev. X* **8**, 041051. 10.1103/PhysRevX.8.041051 (2018).

[27] Clivaz, F. et al. Unifying paradigms of quantum refrigeration: A universal and attainable bound on cooling. *Phys. Rev. Lett.* **123**, 170605. 10.1103/PhysRevLett.123.170605 (2019). 31702237 10.1103/PhysRevLett.123.170605

[28] Brunner, N. et al. Entanglement enhances cooling in microscopic quantum refrigerators. *Phys. Rev. E* **89**, 032115. 10.1103/PhysRevE.89.032115 (2014). 10.1103/PhysRevE.89.032115 24730798

[29] Hewgill, A., Ferraro, A. & De Chiara, G. Quantum correlations and thermodynamic performances of two-qubit engines with local and common baths. *Phys. Rev. A* **98**, 042102. 10.1103/PhysRevA.98.042102 (2018).

[30] Camati, P. A., Santos, J. F. G. & Serra, R. M. Coherence effects in the performance of the quantum otto heat engine. *Phys. Rev. A* **99**, 062103. 10.1103/PhysRevA.99.062103 (2019).

[31] Uzdin, R., Levy, A. & Kosloff, R. Equivalence of quantum heat machines, and quantum-thermodynamic signatures. *Phys. Rev. X* **5**, 031044. 10.1103/PhysRevX.5.031044 (2015).

[32] Peterson, J. P. S. et al. Experimental characterization of a spin quantum heat engine. *Phys. Rev. Lett.* **123**, 240601. 10.1103/PhysRevLett.123.240601 (2019). 31922824 10.1103/PhysRevLett.123.240601

[33] Barrios, G. A., Albarrán-Arriagada, F., Cárdenas-López, F. A., Romero, G. & Retamal, J. C. Role of quantum correlations in light-matter quantum heat engines. *Phys. Rev. A* **96**, 052119. 10.1103/PhysRevA.96.052119 (2017).

[34] Dillenschneider, R. & Lutz, E. Energetics of quantum correlations. *EPL (Europhysics Letters)* **88**, 50003 (2009).

[35] Park, J. J., Kim, K.-H., Sagawa, T. & Kim, S. W. Heat engine driven by purely quantum information. *Phys. Rev. Lett.* **111**, 230402 (2013). 24476235 10.1103/PhysRevLett.111.230402

[36] Brandner, K., Bauer, M., Schmid, M. T. & Seifert, U. Coherence-enhanced efficiency of feedback-driven quantum engines. *New J. Phys.* **17**, 065006 (2015).

[37] Rahav, S., Harbola, U. & Mukamel, S. Heat fluctuations and coherences in a quantum heat engine. *Phys. Rev. A* **86**, 043843 (2012).

[38] Uzdin, R. Coherence-induced reversibility and collective operation of quantum heat machines via coherence recycling. *Phys. Rev. Appl.* **6**, 024004 (2016).

[39] Dorfman, K. E., Xu, D. & Cao, J. Efficiency at maximum power of a laser quantum heat engine enhanced by noise-induced coherence. *Phys. Rev. E* **97**, 042120. 10.1103/PhysRevE.97.042120 (2018). 29758726 10.1103/PhysRevE.97.042120

[40] Doyeux, P., Leggio, B., Messina, R. & Antezza, M. Quantum thermal machine acting on a many-body quantum system: Role of correlations in thermodynamic tasks. *Phys. Rev. E* **93**, 022134 (2016). 26986315 10.1103/PhysRevE.93.022134

[41] Klatzow, J. et al. Experimental demonstration of quantum effects in the operation of microscopic heat engines. *Phys. Rev. Lett.* **122**, 110601. 10.1103/PhysRevLett.122.110601 (2019). 30951320 10.1103/PhysRevLett.122.110601

[42] Chiara, G. D. et al. Reconciliation of quantum local master equations with thermodynamics. *New J. Phys.* **20**, 113024. 10.1088/1367-2630/aaecee (2018).

[43] Brask, J. B., Haack, G., Brunner, N. & Huber, M. Autonomous quantum thermal machine for generating steady-state entanglement. *New J. Phys.* **17**, 113029. 10.1088/1367-2630/17/11/113029 (2015).

[44] Werlang, T., Marchiori, M. A., Cornelio, M. F. & Valente, D. Optimal rectification in the ultrastrong coupling regime. *Phys. Rev. E* **89**, 062109. 10.1103/PhysRevE.89.062109 (2014). 10.1103/PhysRevE.89.062109 25019727

[45] Man, Z.-X., An, N. B. & Xia, Y.-J. Controlling heat flows among three reservoirs asymmetrically coupled to two two-level systems. *Phys. Rev. E* **94**, 042135. 10.1103/PhysRevE.94.042135 (2016). 27841562 10.1103/PhysRevE.94.042135

[46] Hewgill, A., De Chiara, G. & Imparato, A. Quantum thermodynamically consistent local master equations. *Phys. Rev. Res.* **3**, 013165. 10.1103/PhysRevResearch.3.013165 (2021).

[47] Landi, G. T., Poletti, D. & Schaller, G. Nonequilibrium boundary-driven quantum systems: Models, methods, and properties. *Rev. Mod. Phys.* **94**, 045006. 10.1103/RevModPhys.94.045006 (2022).

[48] Man, Z.-X., Tavakoli, A., Brask, J. B. & Xia, Y.-J. Improving autonomous thermal entanglement generation using a common reservoir. *Phys. Scr.* **94**, 075101. 10.1088/1402-4896/ab0c51 (2019).

[49] Manzano, G., Silva, R. & Parrondo, J. M. R. Autonomous thermal machine for amplification and control of energetic coherence. *Phys. Rev. E* **99**, 042135. 10.1103/PhysRevE.99.042135 (2019). 31108722 10.1103/PhysRevE.99.042135

[50] Hammam, K., Hassouni, Y., Fazio, R. & Manzano, G. Optimizing autonomous thermal machines powered by energetic coherence. *New J. Phys.* **23**, 043024. 10.1088/1367-2630/abeb47 (2021).

[51] Wang, Y., Man, Z.-X., Zhang, Y.-J. & Xia, Y.-J. Work costs and operating regimes for different manners of system-reservoir interactions via collision model. *New J. Phys.* **24**, 053030. 10.1088/1367-2630/ac6a01 (2022).

[52] Giovannetti, V. & Palma, G. M. Master equations for correlated quantum channels. *Phys. Rev. Lett.* **108**, 040401. 10.1103/PhysRevLett.108.040401 (2012). 22400814 10.1103/PhysRevLett.108.040401

[53] Lorenzo, S., McCloskey, R., Ciccarello, F., Paternostro, M. & Palma, G. M. Landauer’s principle in multipartite open quantum system dynamics. *Phys. Rev. Lett.* **115**, 120403. 10.1103/PhysRevLett.115.120403 (2015). 26430974 10.1103/PhysRevLett.115.120403

[54] Lorenzo, S., Farace, A., Ciccarello, F., Palma, G. M. & Giovannetti, V. Heat flux and quantum correlations in dissipative cascaded systems. *Phys. Rev. A* **91**, 022121. 10.1103/PhysRevA.91.022121 (2015).

[55] Zhang, Q., Xia, Y.-J. & Man, Z.-X. Effects of one-way correlations on thermodynamics of a multipartite open quantum system. *Phys. Rev. A* **108**, 062211. 10.1103/PhysRevA.108.062211 (2023).

[56] Rau, J. Relaxation phenomena in spin and harmonic oscillator systems. *Phys. Rev.* **129**, 1880. 10.1103/PhysRev.129.1880 (1963).

[57] Ciccarello, F., Lorenzo, S., Giovannetti, V. & Palma, G. M. Quantum collision models: Open system dynamics from repeated interactions. *Phys. Rep.* **954**, 1 (2022).

[58] Man, Z.-X., Xia, Y.-J. & Lo Franco, R. Temperature effects on quantum non-markovianity via collision models. *Phys. Rev. A* **97**, 062104. 10.1103/PhysRevA.97.062104 (2018).

[59] Jin, J. S. & s. Yu, C. Non-markovianity in the collision model with environmental block. *New J. Phys.* **20**, 053026 (2018). 10.1088/1367-2630/aac0cb.

[60] Barra, F. The thermodynamic cost of driving quantum systems by their boundaries. *Sci. Rep.* **5**, 14873. 10.1038/srep14873 (2015). 26445899 10.1038/srep14873 PMC4597202

[61] Lorenzo, S., McCloskey, R., Ciccarello, F., Paternostro, M. & Palma, G. M. Landauer’s principle in multipartite open quantum system dynamics. *Phys. Rev. Lett.* **115**, 120403. 10.1103/PhysRevLett.115.120403 (2015). 26430974 10.1103/PhysRevLett.115.120403

[62] Pezzutto, M., Paternostro, M. & Omar, Y. Implications of non-markovian quantum dynamics for the landauer bound. *New J. Phys.* **18**, 123018. 10.1088/1367-2630/18/12/123018 (2016).

[63] Man, Z.-X., Xia, Y.-J. & Lo Franco, R. Validity of the landauer principle and quantum memory effects via collisional models. *Phys. Rev. A* **99**, 042106. 10.1103/PhysRevA.99.042106 (2019).

[64] Seah, S. et al. Collisional quantum thermometry. *Phys. Rev. Lett.* **123**, 180602. 10.1103/PhysRevLett.123.180602 (2019). 31763916 10.1103/PhysRevLett.123.180602

[65] Buffoni, L., Solfanelli, A., Verrucchi, P., Cuccoli, A. & Campisi, M. Quantum measurement cooling. *Phys. Rev. Lett.* **122**, 070603. 10.1103/PhysRevLett.122.070603 (2019). 30848614 10.1103/PhysRevLett.122.070603

